# Unexpected effects of azole transporter inhibitors on antifungal susceptibility in *Candida glabrata* and other pathogenic *Candida* species

**DOI:** 10.1371/journal.pone.0180990

**Published:** 2017-07-11

**Authors:** Yohsuke Nagayoshi, Taiga Miyazaki, Shintaro Shimamura, Hironobu Nakayama, Asuka Minematsu, Shunsuke Yamauchi, Takahiro Takazono, Shigeki Nakamura, Katsunori Yanagihara, Shigeru Kohno, Hiroshi Mukae, Koichi Izumikawa

**Affiliations:** 1 Department of Infectious Diseases, Nagasaki University Graduate School of Biomedical Sciences, Nagasaki, Japan; 2 Second Department of Internal Medicine, Nagasaki University, Nagasaki, Japan; 3 Faculty of Pharmaceutical Sciences, Suzuka University of Medical Sciences, Mie, Japan; 4 Department of Respiratory Medicine, Nagasaki University Graduate School of Biomedical Sciences, Nagasaki, Japan; 5 Department of Laboratory Medicine, Nagasaki University Graduate School of Biomedical Sciences, Nagasaki, Japan; Leibniz Institute for Natural Products Research and Infection Biology- Hans Knoell Institute, GERMANY

## Abstract

The pathogenic fungus *Candida glabrata* is often resistant to azole antifungal agents. Drug efflux through azole transporters, such as Cdr1 and Cdr2, is a key mechanism of azole resistance and these genes are under the control of the transcription factor Pdr1. Recently, the monoamine oxidase A (MAO-A) inhibitor clorgyline was shown to inhibit the azole efflux pumps, leading to increased azole susceptibility in *C*. *glabrata*. In the present study, we have evaluated the effects of clorgyline on susceptibility of *C*. *glabrata* to not only azoles, but also to micafungin and amphotericin B, using wild-type and several mutant strains. The addition of clorgyline to the culture media increased fluconazole susceptibility of a *C*. *glabrata* wild-type strain, whereas micafungin and amphotericin B susceptibilities were markedly decreased. These phenomena were also observed in other medically important *Candida* species, including *Candida albicans*, *Candida parapsilosis*, *Candida tropicalis*, and *Candida krusei*. Expression levels of *CDR1*, *CDR2* and *PDR1* mRNAs and an amount of Cdr1 protein in the *C*. *glabrata* wild-type strain were highly increased in response to the treatment with clorgyline. However, loss of Cdr1, Cdr2, Pdr1, and a putative clorgyline target (Fms1), which is an ortholog of human MAO-A, or overexpression of *CDR1* did not affect the decreased susceptibility to micafungin and amphotericin B in the presence of clorgyline. The presence of other azole efflux pump inhibitors including milbemycin A4 oxime and carbonyl cyanide 3-chlorophenylhydrazone also decreased micafungin susceptibility in *C*. *glabrata* wild-type, Δ*cdr1*, Δ*cdr2*, and Δ*pdr1* strains. These findings suggest that azole efflux pump inhibitors increase azole susceptibility but concurrently induce decreased susceptibility to other classes of antifungals independent of azole transporter functions.

## Introduction

The pathogenic fungus *Candida glabrata* is the second most common cause of candidemia and is relatively resistant to azole antifungal agents [1]. A key mechanism of azole-resistance is the reduction in the intracellular drug concentration, which is achieved by activation of azole transporters, such as Cdr1 and Cdr2 (formerly denoted Pdh1) [2–4]. Inhibition of these transporters is one effective way for combating azole-resistance. A recent study discovered that the monoamine oxidase A (MAO-A) inhibitor clorgyline inhibits the activity of azole efflux pumps, such as Cdr1, Cdr2, and Mdr1, leading to increased azole susceptibility in *C*. *glabrata* and *Candida albicans* [5]. Clorgyline suppresses the oxidation of human MAO-A and has been used as an antidepressant drug [6], and may be useful for the treatment of heart failure [7] and prostate cancer [8], suggesting that clorgyline might be a candidate for combination with azole antifungals.

Antifungals of the echinocandin class or polyene class have been suggested to be effective for treatment of candidiasis caused by *C*. *glabrata* [9]. However, recent epidemiological surveys have revealed the emergence of increasing numbers of isolates that have decreased susceptibility to echinocandins, particularly among fluconazole resistant isolates [10–12]. Furthermore, it has also been reported that polyene susceptibility is attenuated in azole-resistant isolates [11].

In the present study, we have evaluated the effects of clorgyline on susceptibility of *C*. *glabrata* to not only azoles, but also to echinocandin and polyene antifungals, using wild-type and several mutant strains, including Δ*cdr1* and Δ*cdr2* strains.

## Materials and methods

### Strains and culture conditions

The *C*. *glabrata*, *C*. *albicans*, *C*. *parapsilosis*, *C*. *tropicalis*, and *C*. *krusei* strains used in this study are listed in Table 1. Cells were grown at 30°C in YPD medium (1% yeast extract, 2% peptone, and 2% dextrose; Difco Laboratories, Detroit, MI), minimal medium (MIN) (0.67% yeast nitrogen base without amino acids [Difco Laboratories], 2% dextrose), synthetic complete medium (SC), or SC lacking tryptophan (SC-trp) [13].

**Table 1:** Strains used in this study.

Strain	Genotype or description	Reference or source
CBS138	*Candida glabrata* Wild type	[39]
2001T	Δ*trp1* (a derivative of CBS138)	[40]
2001HT	Δ*his3*, Δ*trp1* (a derivative of CBS138)	[40]
KUE200	Δ*his3*, Δ*trp1* (a derivative of 2001HT)	[41]
TG-C1	Δ*cdr1*:: *TRP1* (made from 2001T)	This study
TG-C2	Δ*cdr2*:: *TRP1* (made from 2001T)	This study
TG-C3	Δ*pdr1*:: *HIS3*, Δ*trp1* (made from KUE200)	This study
TG-C4	Δ*fms1*:: *HIS3*, Δ*trp1* (made from 2001HT)	This study
TG-C5	2001T containing pCgACT-PC1	This study
TG11	2001T containing pCgACT-P	[14]
SC5314	*Candida albicans* wild type	[42]
ATCC 90018	*Candida parapsilosis* wild type	American type culture collection (ATCC), Manassas, VA
ATCC 750	*Candida tropicalis* wild type	ATCC, Manassas, VA
ATCC 6258	*Candida krusei* wild type	ATCC, Manassas, VA

### Plasmid and strain construction

The primers used in this study are listed in Table 2. Sequence information of *C*. *glabrata* genes was obtained from the *Candida* genome database (http://www.candidagenome.org). To construct an overexpression plasmid for *CDR1*, a 4.5-kb BamHI-SalI PCR fragment containing *C*. *glabrata CDR1* open reading frame was inserted into the BamHI-SalI site of pCgACT-P [14] and expressed under the *Saccharomyces cerevisiae PGK1* promoter (pCgACT-PC1).

**Table 2:** Primers used in this study.

For gene deletion	Target gene	Sequence (5'–3') (sequences homologous to flanking regions of the target gene are shown in italics. Sequences shown in bold exist in pBSK-HIS, or pBSK-TRP)
CgCDR1 100-F	*CDR1*	*GGACCTGGACTTGGATTTGGACTTGGAAAAAGTATATAAAAACTATATGCCAATTGATACTTACAGGAAAAAGAATTTACAACTCTTGATATATACAAA***TAATACGACTCACTATAGGGC**
CgCDR1 100-R	*CDR1*	*GATGATAAACAAACATGAACTTAAATTATAATATTAAAAATAAAATCTATGAAATTTATAAAAACTAATAGTTTTCCGAATGCAATATGTATTAATACC***CGCTCTAGAACTAGTGGATCC**
CgCDR2 100-F	*CDR2*	*CTGGAACGTTTATAAGGTTTGTTGGGTGTATTAGGTTGGGAAGTAGGGAACACCACTCAGCTCACCTCAGTAAAACAAATTATTACAACATAACAAATA***TAATACGACTCACTATAGGGC**
CgCDR2 100-R	*CDR2*	*CGGATGTTTACATGAAAAGTCAACCAAACACAATATATTGGTCGATTAGTTCTTTGTTTAGACAGATATGGTTTAATTAAAGTAGCATAAATTGTCAGC***CGCTCTAGAACTAGTGGATCC**
CgPDR1 100-F	*PDR1*	*CTTCGTACCCCATATCGTATTGCCATTGTGATATGGAATTAGTGTTTTATTCTGCCTTTTTTTTTAGAATATATTGGTAAAGTCATTCTTTAGCTACGT***TAATACGACTCACTATAGGGC**
CgPDR1 100-R	*PDR1*	*CTAAGTCTCATGTAAAATTATGCAACATAACCACTAAAAAATGATTTTTCAGATTAAATATAAAATTATACAGGCTATGCACACTGTCTAAATTAATAG***CGCTCTAGAACTAGTGGATCC**
CgFMS1 100-F	*FMS1*	*CGAGAACAAATTAAGAGGCAACGAAGGGAATATACACCCGCCACCCATCAACTGGAAGGGTGAAATCATTTGTAAAAAAAGGCACCAAATATAAAAGAA***TAATACGACTCACTATAGGGC**
CgFMS1 100-R	*FMS1*	*GTATTTAAAGTTCTTTTTTTGTTAATAAATATTCCGACCAAAAATAGCACGCAACGCCATTGTGCATATTTTAAATACAGAAAAAGGAATACGTACAAG***CGCTCTAGAACTAGTGGATCC**
For real-time qRT-PCR	Target gene	Sequence (5’-3’)
Cg18S-F	18S rRNA	TGACTCAACACGGGGAAACTCAC
Cg18S-R	18S rRNA	CACTCCACCAACTAAGAACGGC
CgPDR1-F	*PDR1*	CAATGTCTCAGATACCACCACCG
CgPDR1-R	*PDR1*	TGTCTTTAGAATCCAACTGCGTTTG
CgCDR1-F	*CDR1*	ACCATACTCCCTTGGCTGTTACTG
CgCDR1-R	*CDR1*	TCCTTTGAAGAGGCTGTGGATG
CgCDR2-F	*CDR2*	TCTGAAGACACAGGCACACATTG
CgCDR2-R	*CDR2*	CTGGGCGTCACCAAAATAAATC
CgFMS1-F	*FMS1*	GCTGACACAGAGTGGTAAGAGTTGC
CgFMS1-R	*FMS1*	AAAGAGACGGTTCGTGAGCGTG

*C*. *glabrata* deletion strains were constructed using the one-step PCR-based technique, described previously [15,16]. Briefly, a deletion construct was amplified from either pBSK-HIS (pBluescript II SK+ [Stratagene, La Jolla, CA] containing *C*. *glabrata HIS3* at the XhoI site) or pBSK-TRP (pBluescript II SK+ containing *C*. *glabrata TRP1* at the XhoI site) using primers tagged with 100-bp sequences homologous to the flanking regions of the target open reading frame. *C*. *glabrata* parent strains were subsequently transformed with the deletion construct, and the resulting transformants were selected by tryptophan or histidine prototrophy, as appropriate [15]. Successful homologous recombination was verified by diagnostic PCR and the absence of mRNA expression of the target genes was also confirmed by real-time quantitative reverse transcription PCR (real-time qRT-PCR) (data not shown). Transformation of *C*. *glabrata* was performed using the lithium acetate protocol, as described previously [17]. *CDR1*-overexpressed and its control strains were constructed by transformation of 2001T with pCgACT-PC1 and pCgACT-P, respectively, selected by tryptophan prototrophy, and verified by immunoblotting.

### Compounds

Clorgyline (Sigma Aldrich, St. Louis, MO) was dissolved in distilled water and stored at 4°C. Fluconazole (Sigma Aldrich) was dissolved in dimethyl sulfoxide (DMSO), and stored at 4°C. Amphotericin B (Sigma Aldrich), carbonyl cyanide 3-chlorophenylhydrazone (CCCP; Sigma Aldrich) and milbemycin A4 oxime (Novartis Animal Health, Basel, Switzerland) was dissolved in DMSO and stored at -20°C. Micafungin (Astellas, Tokyo, Japan) was dissolved in distilled water and stored at -20°C.

### Susceptibility test

To examine the dose-dependence of the effects of clorgyline on fluconazole susceptibility, a checkerboard assay was performed in triplicate, in three independent experiments. MIN was used instead of RPMI, because RPMI broth mixed with a high concentration of clorgyline became turbid. The effects of clorgyline on micafungin and amphotericin B susceptibility were also examined by checkerboard assays using SC broth buffered to pH7.0. Two-fold dilutions of each compound were aliquoted into a 96-well plate. Each well was then inoculated with cell suspension at the final concentration of 5 × 10^2^ cells/well. Plates were incubated at 35°C for 48 h. Cell growth was monitored using an absorption spectrometer at 620 nm. Minimum drug concentration that inhibits growth by more than 80% relative to drug free control was defined as minimum inhibitory concentration (MIC). The fractional inhibitory concentration (FIC) was calculated by the following formula: FIC for drug A = MIC of drug A in combination with drug B/MIC of drug A alone. The sum of FIC for drug A and FIC for drug B was defined as FIC index (FICI). Drug interaction was classified as synergistic if its FICI was 0.5 or less than 0.5, and was classified as antagonistic if its FICI was greater than 4, as described previously [18].

A spot dilution test was carried out as described previously [14]. Briefly, logarithmic-phase cells grown in MIN broth or SC broth were harvested and adjusted to the concentration of 2 × 10^7^ cells/ml. Serial 10-fold dilutions were then prepared, and 5 μl of each dilution was spotted onto an agar plate containing a test compound at the desired concentration. Plates were incubated at 30°C for 48 h.

### Immunoblotting

Anti-Cdr1 and Cdr2 antibodies were kindly provided by Dr. Dominique Sanglard. Anti-Pgk1 (OriGene EU, Herford, Germany) and rabbit IgG-hrp (GE Healthcare, Pittsburgh, PA) antibodies were purchased. Liquid-cultured *C*. *glabrata* strains at logarithmic-phase were harvested and lysed using Minute Total Protein Extraction Kit for Microbes with Thick Cell Walls (Invent Biotechnologies, Plymouth, MN) according to its instruction. Lysates were separated by SDS–PAGE, and transferred to a polyvinylidene difluoride membrane (BIO-RAD, Hercules, CA). Each protein was detected using the indicated antibodies, an enhanced chemiluminescent substrate (Thermo Fisher Scientific, Waltham, MA) and ChemiDoc Touch imaging system (BIO-RAD).

### Real-time qRT-PCR

To examine expression levels of the genes associated with azole efflux, as well as *FMS1*, logarithmic-phase cells grown in YPD broth were adjusted to 2 × 10^7^ cells/ml and then further incubated in the presence of 80 μg/ml of clorgyline for up to 4 h. Total RNA was extracted at the indicated time points, using a FastRNA Pro Red Kit (Qbiogene, Carlsbad, CA).

Real-time qRT-PCR was then performed as described previously [19]. Briefly, first strand cDNA was synthesized with a QuantiTect Reverse Transcription kit (Qiagen, Valencia, CA), using 1 μg of total RNA as template, in a final volume of 20 μl, and 3 μl of the resulting cDNA was then used as the template for individual PCRs. These reactions were performed using gene-specific primers (Table 2) and a QuantiTect SYBR Green PCR kit (Qiagen). Real-time qRT-PCR was performed using a 7500 Real-Time PCR System (Applied Biosystems, Foster City, CA). Extraction of RNA and real-time qRT-PCR were performed in triplicate, in three independent experiments.

## Results and discussion

### Clorgyline improves fluconazole-susceptibility in *C*. *glabrata*

**Fig 1:**
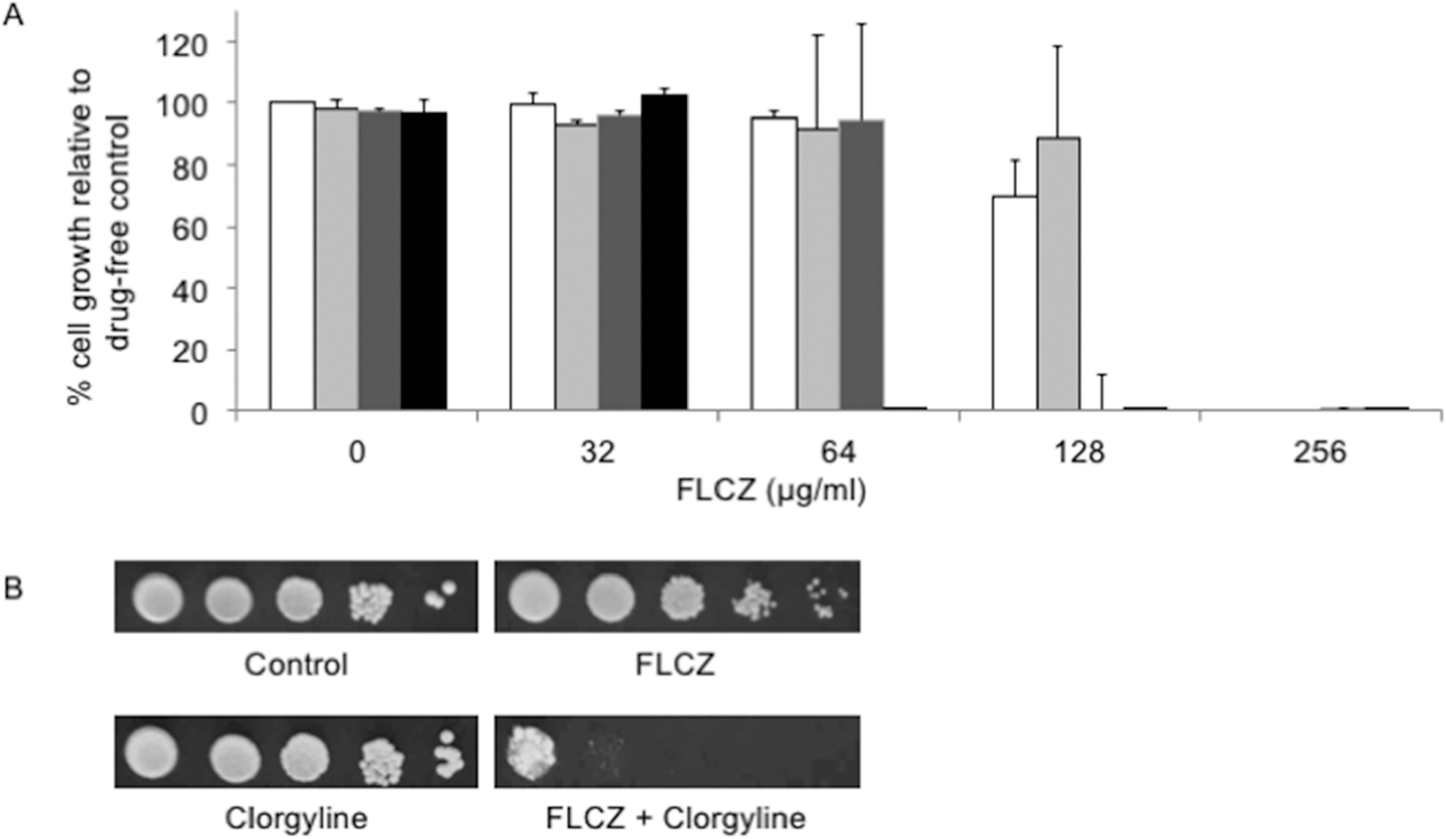
(A) The effect of clorgyline on fluconazole susceptibility in the *C*. *glabrata* CBS138 strain. Checkerboard susceptibility assay was performed as described in the Materials and Methods section. Percentages of cell growth relative to drug-free control are expressed as means ± SE. Clorgyline concentration: white bar, 0 μg/ml; light grey bar, 40 μg/ml; dark grey bar, 80 μg/ml; and black bar, 160 μg/ml. (B) Spot dilution assay. Logarithmic-phase cells were adjusted to 2 × 10^7^ cells/ml, and 5 μl of serial 10-fold dilutions were then spotted onto SC plates containing FLCZ, clorgyline, or both. Plates were incubated at 30°C for 48 h. Final concentrations: clorgyline, 80 μg/ml; and FLCZ, 64 μg/ml.

In a previous study, the chemosensitizing effect of clorgyline to fluconazole in *C*. *glabrata* was demonstrated by a disk diffusion assay, and the clorgyline MIC in *C*. *glabrata* was reported to exceed 40 μg/ml [5]. To test the effect of clorgyline at a concentration higher than 40 μg/ml on growth in *C*. *glabrata*, and to test the effect of clorgyline on the fluconazole MIC, we performed a checkerboard assay using serial 2-fold dilutions of these two compounds. At 48 h, clorgyline did not inhibit the growth of *C*. *glabrata* at a concentration of up to 160 μg/ml, but at 320 μg/ml, clorgyline inhibited the growth slightly; thus, the MIC of clorgyline may exceed 320 μg/ml. The MIC of fluconazole for *C*. *glabrata* was 256 μg/ml, whereas this decreased to 64 μg/ml in the presence of 160 μg/ml clorgyline (Fig 1A). On the other hand, the MIC of clorgyline for *C*. *glabrata* decreased to 80 μg/ml in the presence of 128μg/ml fluconazole. According to these data, the FICI of these two drugs are less than 0.5, indicating synergism. This effect of clorgyline on fluconazole susceptibility was also supported by spot dilution test (Fig 1B).

The lowest concentration of clorgyline that elicits a chemosensitizing effect on fluconazole was 80 μg/ml. This concentration was sufficient to inhibit azole transporters in the *C*. *glabrata* wild-type strain. We therefore used 80 μg/ml clorgyline in the subsequent experiments, although further evaluation may be needed to detect a more appropriate dose in other *Candida* species.

### Micafungin- and amphotericin-B-susceptibility is markedly decreased in the presence of clorgyline in *Candida* strains

**Fig 2:**
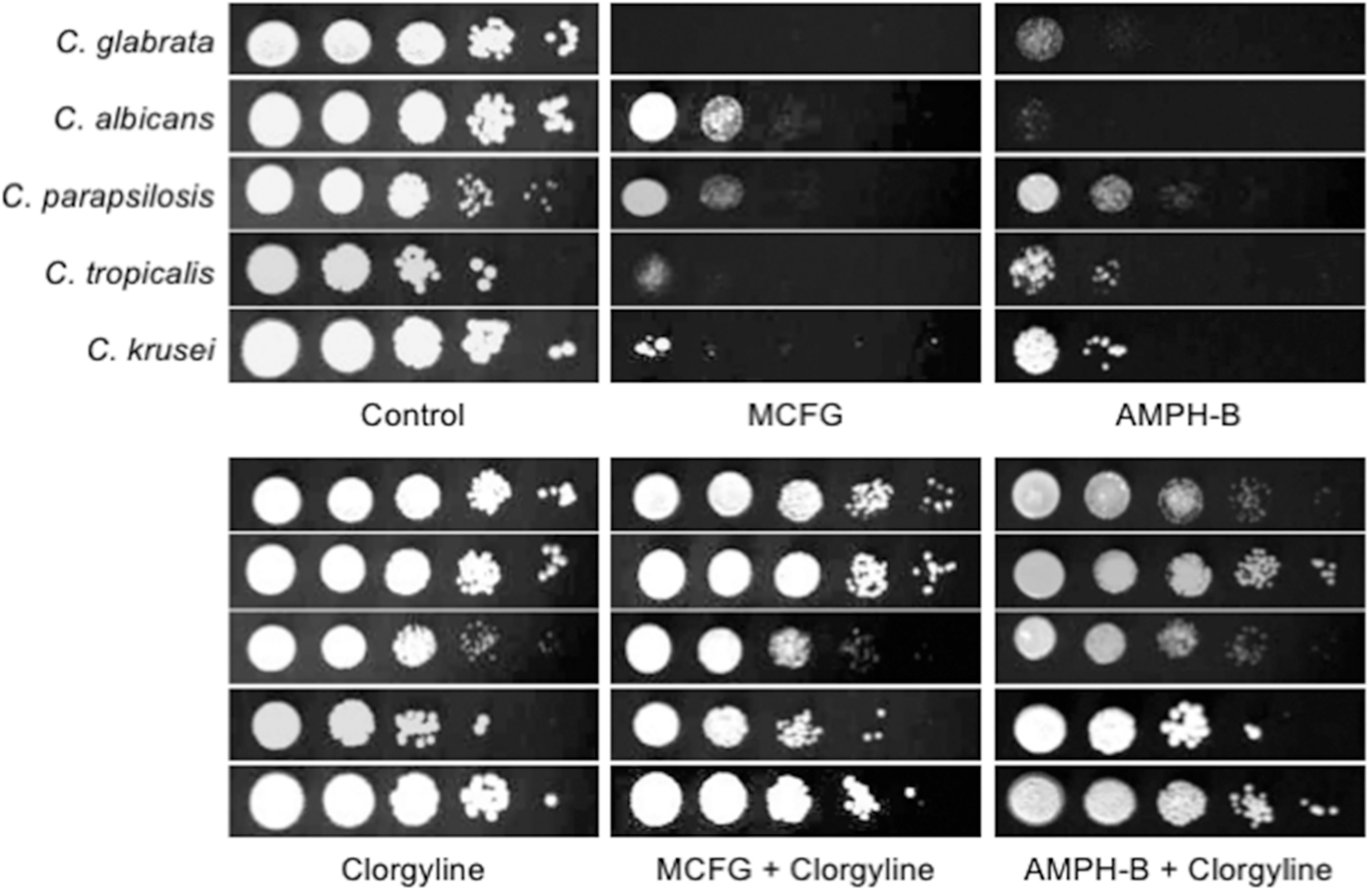
Effects of clorgyline on susceptibility to micafungin and amphotericin B in *Candida* wild-type strains. Spot dilution tests were performed using MIN plates as described in the legend of Fig 1B. Clorgyline was added at a final concentration of 0 μg/ml or 80 μg/ml. Micafungin (MCFG) final concentrations: *C*. *glabrata*, 0.06 μg/ml; *C*. *albicans*, 0.08 μg/ml; *C*. *parapsilosis*, 0.25 μg/ml; *C*. *tropicalis*, 0.06 μg/ml; and *C*. *krusei*, 0.08 μg/ml. Amphotericin B (AMPH-B) final concentrations: *C*. *glabrata*, 0.5 μg/ml; *C*. *albicans*, 0.5 μg/ml; *C*. *parapsilosis*, 1 μg/ml; *C*. *tropicalis*, 0.5 μg/ml; and *C*. *krusei*, 2 μg/ml. *Candida* wild-type strains: *C*. *glabrata*, CBS138; *C*. *albicans*, SC5314; *C*. *parapsilosis*, ATCC 90018; *C*. *tropicalis*, ATCC 750; and *C*. *krusei*, ATCC 6258.

As reported previously, echinocandin and amphotericin B MIC levels for fluconazole-resistant *Candida* isolates tend to be higher than those for fluconazole-susceptible isolates [11,12]. In some reports, it was suggested that overexpression of an azole transporter, particularly Cdr2p, may slightly decrease echinocandin susceptibility in *C*. *albicans* [20–22]. Therefore, we examined whether the azole transporter inhibitor clorgyline affects susceptibility to micafungin and amphotericin B in main *Candida* species including *C*. *glabrata*, *C*. *albicans*, *C*. *parapsilosis*, *C*. *tropicalis*, and *C*. *krusei*, which account for about 95% of clinical isolates of *Candida* species [23]. Addition of 80 μg/ml of clorgyline to a MIN plate did not inhibit any of the strains tested in this study. Contrary to our expectations, clorgyline markedly decreased micafungin- and amphotericin B-susceptibility (Fig 2). In some strains, antagonistic effects of clorgyline with micafungin (Table 3) and amphotericin B (Table 4) were observed as high FICI that exceeded 4 in checkerboard assays, while the other strains also showed slightly decreased susceptibility to micafungin or amphotericin B in the presence of clorgyline. The attenuation of micafungin and amphotericin B activity by this agent has never been reported. If this phenomenon is mediated via inhibition of an azole efflux pump, the strategy of chemosensitization by other efflux pump inhibitors may lead to an adverse effect when treatment is switched from azoles to other antifungals, such as echinocandins and polyenes.

**Table 3:** Interaction of micafungin and clorgyline against *Candida* species determined by fractional inhibitory concentration index.

Strain	MIC_80_ (μg/mL)			FICI
	MCFG	Clor	MCFG/Clor	
*C*. *glabrata* CBS138	0.06	80	0.25/80	5
*C*. *albicans* SC5314	0.06	80	0.25/80	5
*C*. *tropicalis* ATCC 750	0.06	80	0.25/80	5
*C*. *krusei* ATCC 6258	0.12	80	0.25/80	3
*C*. *parapsilosis* ATCC 90018	1	40	2/40	3

Note: MCFG, micafungin; Clor, clorgyline; FICI, fractional inhibitory concentration index.

**Table 4:** Interaction of amphotericin B and clorgyline against *Candida* species determined by fractional inhibitory concentration index.

Strain	MIC_80_ (μg/mL)			FICI
	AMPH-B	Clor	AMPH-B/Clor	
*C*. *glabrata* CBS138	2	80	4/20	2.25
*C*. *albicans* SC5314	1	80	4/40	4.5
*C*. *tropicalis* ATCC 750	2	80	>4/40	4.5
*C*. *krusei* ATCC 6258	2	80	>4/80	5
*C*. *parapsilosis* ATCC 90018	>4	40	>4/40	2

Note: AMPH-B, amphotericin B; Clor, clorgyline; FICI, fractional inhibitory concentration index.

### The effect of clorgyline on micafungin- and amphotericin B- susceptibility may not be caused directly by azole transporter inhibition in *C*. *glabrata*

**Fig 3:**
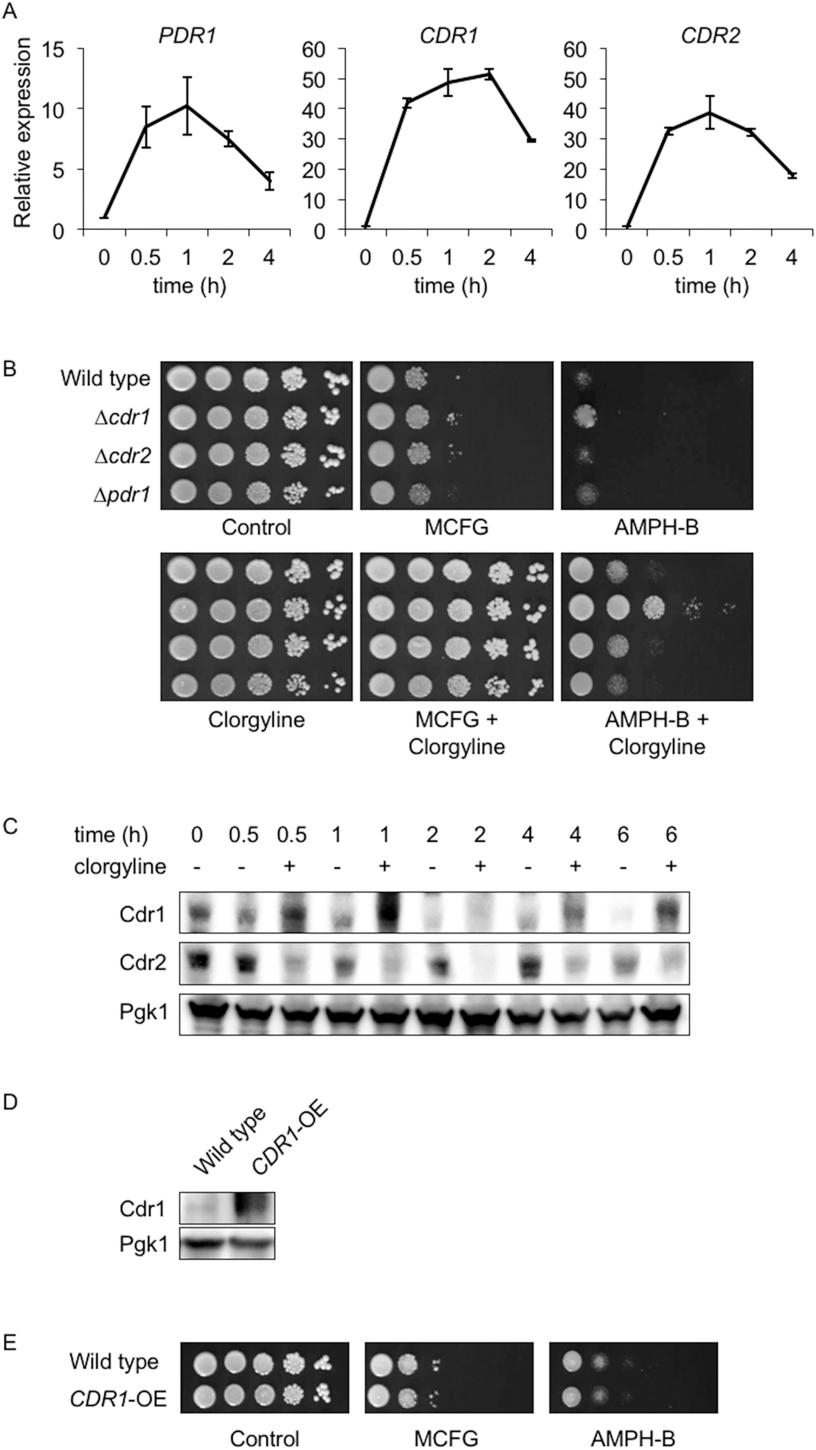
(A) Time-course analysis of *PDR1*, *CDR1*, and *CDR2* expression in the *C*. *glabrata* wild-type strain. Logarithmic-phase cells were prepared in YPD broth and clorgyline was added at the concentration of 80 μg/ml. Cells were incubated at 30°C with agitation and total RNAs were extracted at indicated time points. *PDR1*, *CDR1*, and *CDR2* mRNA abundance was measured by real-time qRT-PCR and normalized by using 18S rRNA as an internal control. Data were expressed as expression ratio relative to the mRNA abundance immediately before the clorgyline addition (time point 0). The mean ± SE of three independent experiments are shown. (B) Effects of clorgyline on susceptibility to micafungin and amphotericin B in *C*. *glabrata* wild-type strain and *CDR1*, *CDR2*, and *PDR1* mutant strains. Spot dilution tests were performed as described in the legend of Fig 1B. Final drug concentrations: clorgyline, 80 μg/ml; micafungin (MCFG), 0.03 μg/ml; and amphotericin B (AMPH-B), 1.25 μg/ml. *C*. *glabrata* strains: wild-type, CBS138; Δ*cdr1*, TG-C1; Δ*cdr2*, TG-C2; and Δ*pdr1*, TG-C3. (C) Time-course analysis of Cdr1 and Cdr2 expression in the *C*. *glabrata* wild-type strain CBS 138. Logarithmic-phase cells were incubated at 30°C with 80 μg/ml of clorgyline in YPD broth. Total proteins were extracted at indicated time points, separated by SDS-PAGE, and blotted to a polyvinylidene difluoride membrane. Each protein was detected using the indicated antibodies. (D) Expression of Cdr1 protein in the wild-type control and *CDR1*-oversepressing (*CDR1*-OE) strains evaluated by western blot analysis as described above. *C*. *glabrata* strains: Wild type, TG11; and *CDR1*-OE, TG-C5. (E) Micafungin and amphotericin B susceptibility of the *CDR1*-oversepressing (*CDR1*-OE) strain. Spot dilution tests were performed using SC-trp plates as described in the legend of Fig 1B. Final drug concentrations: clorgyline, 80 μg/ml; micafungin (MCFG), 0.025 μg/ml; and amphotericin B (AMPH-B), 1.25 μg/ml. *C*. *glabrata* strains: Wild-type, TG11; and *CDR1*-OE, TG-C5.

A previous report has shown that deletion of genes encoding azole efflux pumps, such as *CDR1*, *CDR2*, and *MDR1*, does not alter caspofungin susceptibility in *C*. *albicans* [24]. However overexpression of these pumps slightly decreases echinocandin susceptibility [21,22]. It is still unclear whether the azole transporters Cdr1 and Cdr2 are involved in the efflux of echinocandins. It has previously been shown that clorgyline inhibits rhodamine 6G efflux in a recombinant *Saccharomyces cerevisiae* strain expressing *C*. *albicans CDR1* and *CDR2* [5]. Although rhodamine 6G is a known substrate of Cdr1 and Cdr2 in both *C*. *albicans* and *C*. *glabrata* [25], the precise mechanism by which clorgyline inhibits these azole transporters is not clear. To investigate the clorgyline-mediated inhibition of azole transporters at the mRNA expression level, we performed real-time qRT-PCR. As shown in Fig 3A, clorgyline did not inhibit mRNA expression of *C*. *glabrata CDR1*, *CDR2*, or *PDR1*, but markedly elevated the expression of these genes.

It may be possible that clorgyline alters the substrate specificity of azole transporters, and that activation of Cdr1 or Cdr2, or Pdr1 are involved in transport of echinocandins and polyenes. To investigate this hypothesis, we constructed *C*. *glabrata* Δ*cdr1*, Δ*cdr2*, and Δ*pdr1* strains and compared the micafungin- and amphotericin B-susceptibility of these strains in the presence and absence of clorgyline. The Δ*cdr1* and Δ*pdr1* strains showed increased fluconazole susceptibility, but the susceptibility of Δ*cdr2* strain was equal to the wild-type strain (data not shown), in accordance with previous reports [26,27]. As shown in Fig 3B, deletion of *C*. *glabrata CDR1*, *CDR2*, and *PDR1* did not change the clorgyline effect on micafungin- and amphotericin B- susceptibility.

We also performed western blot analysis to investigate the clorgyline-mediated effect on Cdr1 and Cdr2 protein expression. The treatment with clorgyline increased not only *CDR1* mRNA expression (Fig 3A) but also the amount of Cdr1 protein (Fig 3C) in *C*. *glabrata*. On the other hand, the amount of Cdr2 protein was decreased in the presence of clorgyline for unknown reasons (Fig 3C). In *C*. *albicans*, Cdr1 plays a role in maintaining membrane asymmetry and alterations in membrane lipid composition affects Cdr1 functions [28,29]. In addition, altered sphingolipid biosynthesis resulting in the accumulation of certain long-chain bases affects echinocandin susceptibility in *C*. *albicans* and *C*. *glabrata* [30,31]. Therefore, a possible hypothesis is that decreased susceptibility to micafungin and amphotericin B in the presence of clorgyline may be due to downstream effects of Cdr1 overproduction on membrane composition. To evaluate the effect of overproduction of Cdr1 protein on micafungin and amphotericin B susceptibility, we constructed a *CDR1*-overexpressing strain in *C*. *glabrata* and an increase in the amount of Cdr1 protein was confirmed by western blot analysis (Fig 3D). As shown in Fig 3E, overexpression of *CDR1* did not affect the micafungin- and amphotericin B- susceptibility.

These results suggest that clorgyline decreases the micafungin- and amphotericin B- susceptibility independent of the functions of Cdr1, Cdr2, and Pdr1.

### Function of *C*. *glabrata* Fms1, a putative ortholog of human MAO-A, does not affect the decreased susceptibility to micafungin and amphotericin B in the presence of clorgyline

**Fig 4:**
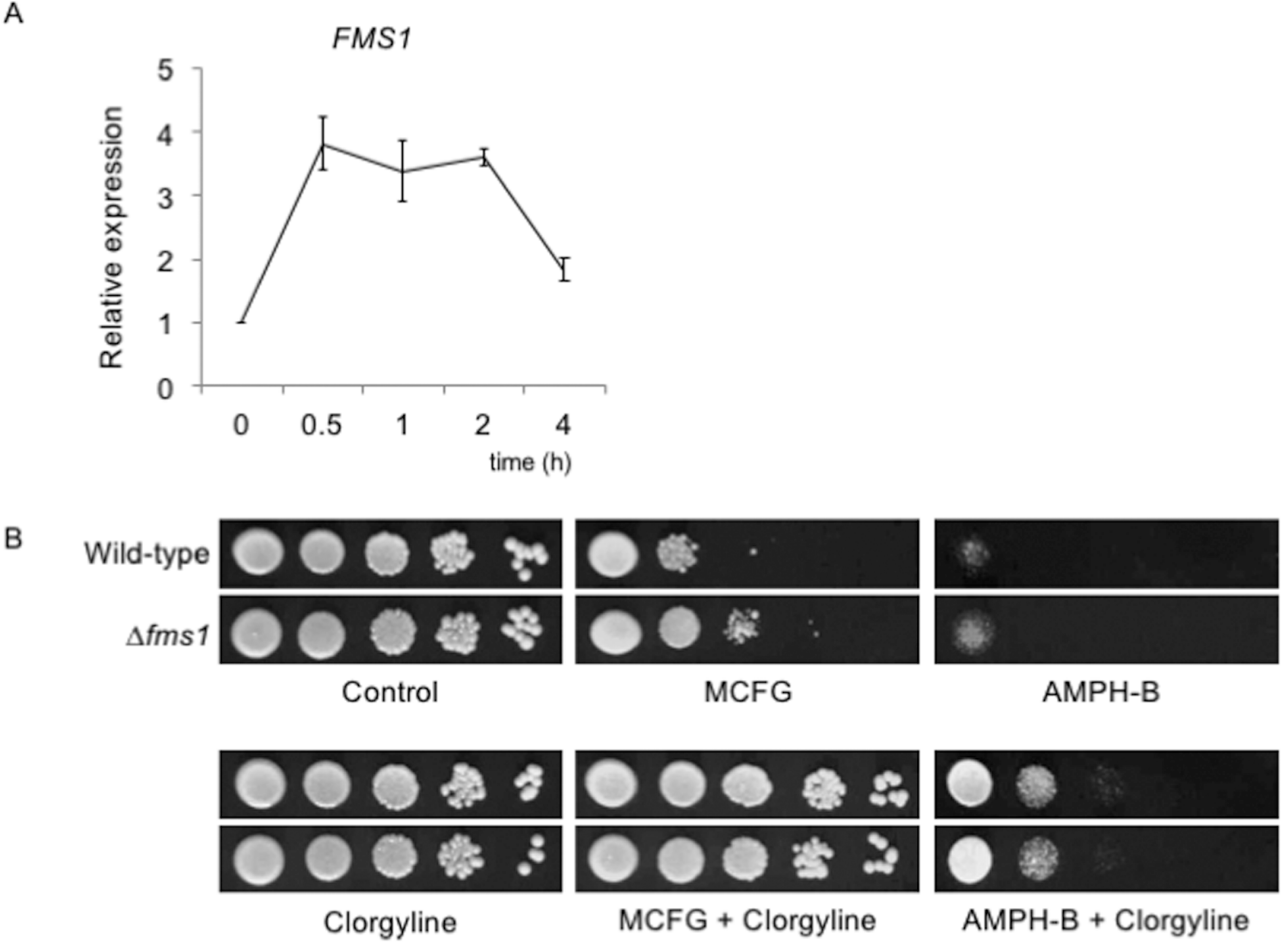
(A) Time-course analysis of *FMS1* expression in *C*. *glabrata* wild-type strain. Cell culture, RNA extraction, real-time qRT-PCR, and data presentation were performed as described in the Materials and Methods section and Fig 3A legend. (B) Effects of clorgyline on susceptibility to micafungin and amphotericin B in the *C*. *glabrata* wild-type and *FMS1* mutant strains. Spot dilution tests were performed as described in the legend of Fig 1B. Final drug concentrations: clorgyline, 80 μg/ml; micafungin (MCFG), 0.03 μg/ml; and amphotericin B (AMPH-B), 1.25 μg/ml. *C*. *glabrata* strains: Wild type, CBS138; and Δ*fms1*, TG-C4.

The molecular target of clorgyline in humans is MAO-A, and its homolog in *C*. *albicans* is *CBP1*, encoding a corticosteroid-binding protein [5,32]. It has previously been suggested that clorgyline may act on Cbp1 and affect plasma membrane proteins, such as efflux pumps [5]. *S*. *cerevisiae* Fms1 is a homolog of *C*. *albicans* Cbp1, and is involved in sterol synthesis [33]. The pairwise alignment of deduced amino acid sequences of the *C*. *glabrata FMS1* ortholog (CAGL0M07612g) and *S*. *cerevisiae FMS1* exhibited 42% identity and 57% similarity (ClustalW alignment with MacVector software version 15.1). Real-time qRT-PCR showed that addition of clorgyline increased mRNA levels of *FMS1* in the *C*. *glabrata* wild-type strain (Fig 4A). Next, we generated a strain lacking *C*. *glabrata FMS1* and evaluated its susceptibility to micafungin and amphotericin B in the presence and absence of clorgyline. As shown in Fig 4B, the effect of clorgyline on micafungin- and amphotericin B- susceptibility was not attenuated in the Δ*fms1* strain, compared to its parental strain, suggesting a possibility that clorgyline may have other target molecules in *C*. *glabrata*.

### Azole transporter inhibitors other than clorgyline also decrease micafungin susceptibility of *C*. *glabrata*

**Fig 5:**
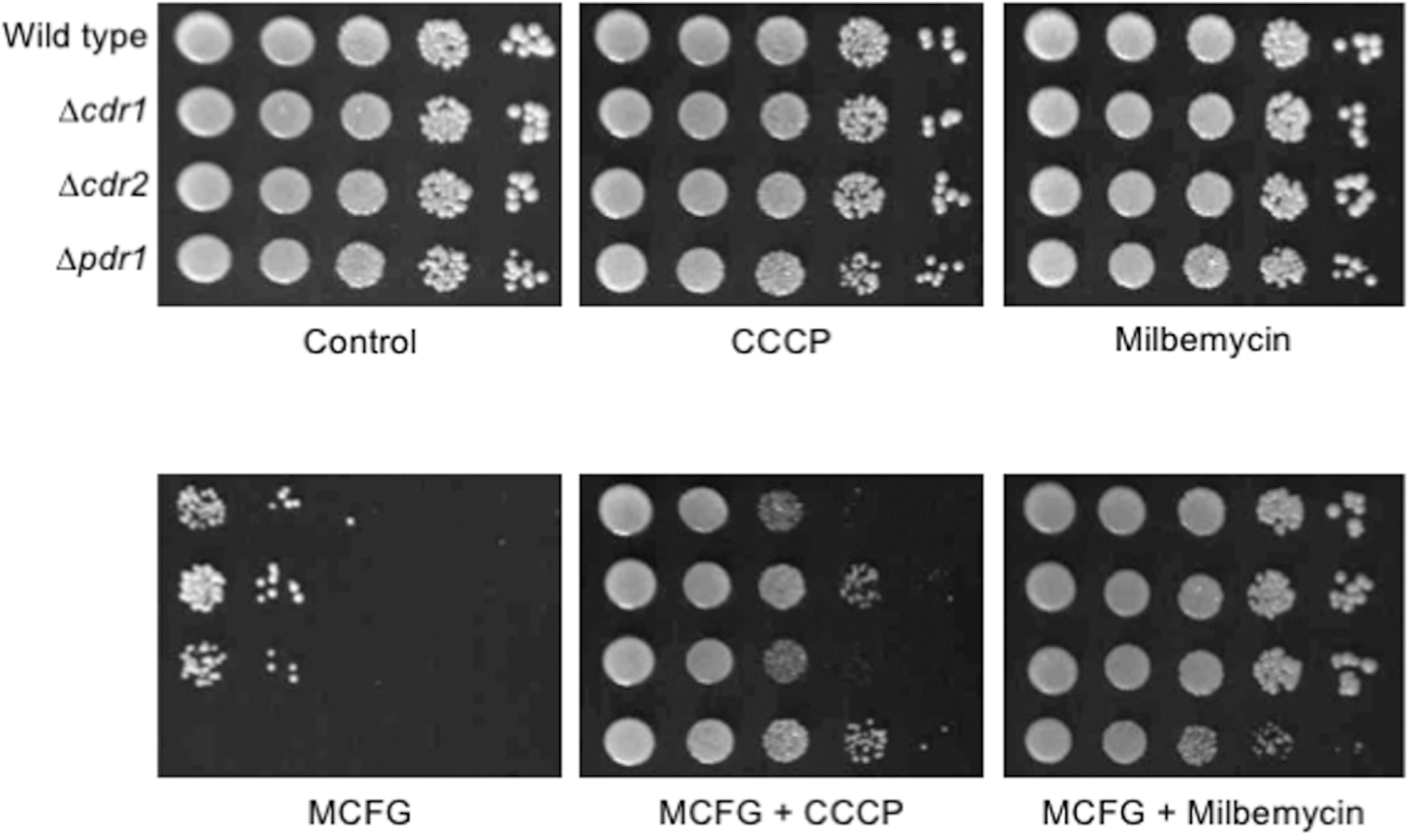
Effects of two known azole transporter inhibitors, carbonyl cyanide 3-chlorophenylhydrazone and milbemycin A4 oxime, on micafungin susceptibility in *C*. *glabrata* wild-type strain and *CDR1*, *CDR2*, and *PDR1* mutant strains. Spot dilution tests were performed as described in the legend of Fig 1B. Final drug concentrations: carbonyl cyanide 3-chlorophenylhydrazone (CCCP), 40 μM; milbemycin A4 oxime (Milbemycin), 4 μM; micafungin (MCFG), 0.03 μg/ml. *C*. *glabrata* strains: Wild type, CBS138; Δ*cdr1*, TG-C1; Δ*cdr2*, TG-C2; and Δ*pdr1*, TG-C3.

The above findings indicated that the effect of clorgyline on micafungin susceptibility may not be caused by efflux of micafungin by an azole transporter. It is possible that clorgyline may directly interfere with the activity of micafungin, without a cellular response. To investigate this, we employed an efflux pump inhibitor other than clorgyline, such as milbemycin [25,34] and CCCP [35–38], and tested their effects on micafungin susceptibility in *C*. *glabrata*. Neither of these agents affected the control growth of any of the strains. Similarly to clorgyline, these two agents also decreased micafungin susceptibility, regardless of the presence or absence of *CDR1*, *CDR2*, and *PDR1* (Fig 5).

As all three azole transporter inhibitors attenuated the micafungin susceptibility, it seems unlikely that all three these compounds may similarly interact with micafungin. We cannot completely discount the involvement of azole transporters in micafungin susceptibility, because we have not yet investigated phenotype of a Δ*cdr1* Δ*cdr2* double deletion strain. Furthermore, the function of upstream regulators of azole transporters remains to be investigated.

## Conclusion

In this study, we observed unexpected effects of clorgyline on antifungal susceptibility in the 5 most common species of *Candida*. Fluconazole susceptibility was increased but susceptibilities to micafungin and amphotericin B were decreased in the presence of azole transporter inhibitors, including clorgyline, CCCP, and milbemycin A4 oxime, in *C*. *glabrata*, independently of functions of azole efflux pumps. Although the details of the underlying molecular mechanism and clinical relevance remain to be studied, our results may provide a clue to the discovery of a novel mechanism of micafungin- and amphotericin B-resistance in pathogenic fungi.

## References

[1] Pfaller MA, Diekema DJ (2007) Epidemiology of invasive candidiasis: a persistent public health problem. Clin Microbiol Rev 20: 133–163. doi: 10.1128/CMR.00029-06 17223626 10.1128/CMR.00029-06 PMC1797637

[2] Bennett JE, Izumikawa K, Marr KA (2004) Mechanism of increased fluconazole resistance in *Candida glabrata* during prophylaxis. Antimicrob Agents Chemother 48: 1773–1777. doi: 10.1128/AAC.48.5.1773-1777.2004 15105134 10.1128/AAC.48.5.1773-1777.2004 PMC400565

[3] Sanglard D, Ischer F, Calabrese D, Majcherczyk PA, Bille J (1999) The ATP binding cassette transporter gene *CgCDR1* from *Candida glabrata* is involved in the resistance of clinical isolates to azole antifungal agents. Antimicrob Agents Chemother 43: 2753–2765. 10543759 10.1128/aac.43.11.2753 PMC89555

[4] Miyazaki H, Miyazaki Y, Geber A, Parkinson T, Hitchcock C, Falconer DJ, et al (1998) Fluconazole resistance associated with drug efflux and increased transcription of a drug transporter gene, *PDH1*, in *Candida glabrata*. Antimicrob Agents Chemother 42: 1695–1701. 9661006 10.1128/aac.42.7.1695 PMC105668

[5] Holmes AR, Keniya MV, Ivnitski-Steele I, Monk BC, Lamping E, Sklar LA, et al (2012) The monoamine oxidase A inhibitor clorgyline is a broad-spectrum inhibitor of fungal ABC and MFS transporter efflux pump activities which reverses the azole resistance of *Candida albicans* and *Candida glabrata* clinical isolates. Antimicrob Agents Chemother 56: 1508–1515. doi: 10.1128/AAC.05706-11 22203607 10.1128/AAC.05706-11 PMC3294898

[6] Bortolato M, Chen K, Shih JC (2008) Monoamine oxidase inactivation: from pathophysiology to therapeutics. Adv Drug Deliv Rev 60: 1527–1533. doi: 10.1016/j.addr.2008.06.002 18652859 10.1016/j.addr.2008.06.002 PMC2630537

[7] Kaludercic N, Takimoto E, Nagayama T, Feng N, Lai EW, Bedja D, et al (2010) Monoamine oxidase A-mediated enhanced catabolism of norepinephrine contributes to adverse remodeling and pump failure in hearts with pressure overload. Circ Res 106: 193–202. doi: 10.1161/CIRCRESAHA.109.198366 19910579 10.1161/CIRCRESAHA.109.198366 PMC2804073

[8] Flamand V, Zhao H, Peehl DM (2010) Targeting monoamine oxidase A in advanced prostate cancer. J Cancer Res Clin Oncol 136: 1761–1771. doi: 10.1007/s00432-010-0835-6 20204405 10.1007/s00432-010-0835-6 PMC2945406

[9] Pappas PG, Kauffman CA, Andes D, Benjamin DK Jr., Calandra TF, Edwards JE Jr., et al (2009) Clinical practice guidelines for the management of candidiasis: 2009 update by the Infectious Diseases Society of America. Clin Infect Dis 48: 503–535. doi: 10.1086/596757 19191635 10.1086/596757 PMC7294538

[10] Pfaller MA, Castanheira M, Lockhart SR, Ahlquist AM, Messer SA, Jones RN. (2012) Frequency of decreased susceptibility and resistance to echinocandins among fluconazole-resistant bloodstream isolates of *Candida glabrata*. J Clin Microbiol 50: 1199–1203. doi: 10.1128/JCM.06112-11 22278842 10.1128/JCM.06112-11 PMC3318516

[11] Mario DA, Denardi LB, Bandeira LA, Antunes MS, Santurio JM, Severo LC, et al (2012) The activity of echinocandins, amphotericin B and voriconazole against fluconazole-susceptible and fluconazole-resistant Brazilian *Candida glabrata* isolates. Mem Inst Oswaldo Cruz 107: 433–436. 22510843 10.1590/s0074-02762012000300022

[12] Posteraro B, Sanguinetti M, Fiori B, La Sorda M, Spanu T, Sanglard D, et al (2006) Caspofungin activity against clinical isolates of azole cross-resistant *Candida glabrata* overexpressing efflux pump genes. J Antimicrob Chemother 58: 458–461. doi: 10.1093/jac/dkl237 16757500 10.1093/jac/dkl237

[13] Kaiser C, Michaelis S, Mitchell A (1994) Laboratory Course Manual for Methods in Yeast Genetics. Cold Spring Harbor, New York: Cold Spring Harbor Laboratory Press.

[14] Miyazaki T, Inamine T, Yamauchi S, Nagayoshi Y, Saijo T, Izumikawa K, et al (2010) Role of the Slt2 mitogen-activated protein kinase pathway in cell wall integrity and virulence in *Candida glabrata*. FEMS Yeast Res 10: 343–352. doi: 10.1111/j.1567-1364.2010.00611.x 20214686 10.1111/j.1567-1364.2010.00611.x

[15] Miyazaki T, Izumikawa K, Nagayoshi Y, Saijo T, Yamauchi S, Morinaga Y, et al (2011) Functional characterization of the regulators of calcineurin in *Candida glabrata*. FEMS Yeast Res 11: 621–630. doi: 10.1111/j.1567-1364.2011.00751.x 22093746 10.1111/j.1567-1364.2011.00751.x

[16] Miyazaki T, Yamauchi S, Inamine T, Nagayoshi Y, Saijo T, Izumikawa K, et al (2010) Roles of calcineurin and Crz1 in antifungal susceptibility and virulence of *Candida glabrata*. Antimicrob Agents Chemother 54: 1639–1643. doi: 10.1128/AAC.01364-09 20100876 10.1128/AAC.01364-09 PMC2849377

[17] Cormack BP, Falkow S (1999) Efficient homologous and illegitimate recombination in the opportunistic yeast pathogen *Candida glabrata*. Genetics 151: 979–987. 10049916 10.1093/genetics/151.3.979 PMC1460538

[18] Johnson MD, MacDougall C, Ostrosky-Zeichner L, Perfect JR, Rex JH (2004) Combination antifungal therapy. Antimicrob Agents Chemother 48: 693–715. doi: 10.1128/AAC.48.3.693-715.2004 14982754 10.1128/AAC.48.3.693-715.2004 PMC353116

[19] Miyazaki T, Nakayama H, Nagayoshi Y, Kakeya H, Kohno S (2013) Dissection of Ire1 functions reveals stress response mechanisms uniquely evolved in *Candida glabrata*. PLoS Pathog 9: e1003160 doi: 10.1371/journal.ppat.1003160 23382685 10.1371/journal.ppat.1003160 PMC3561209

[20] Richards TS, Oliver BG, White TC (2008) Micafungin activity against *Candida albicans* with diverse azole resistance phenotypes. J Antimicrob Chemother 62: 349–355. doi: 10.1093/jac/dkn156 18436555 10.1093/jac/dkn156 PMC2532560

[21] Schuetzer-Muehlbauer M, Willinger B, Krapf G, Enzinger S, Presterl E, Kuchler K (2003) The *Candida albicans* Cdr2p ATP-binding cassette (ABC) transporter confers resistance to caspofungin. Mol Microbiol 48: 225–235. 12657057 10.1046/j.1365-2958.2003.03430.x

[22] Niimi K, Maki K, Ikeda F, Holmes AR, Lamping E, Niimi M, et al (2006) Overexpression of *Candida albicans CDR1*, *CDR2*, or *MDR1* does not produce significant changes in echinocandin susceptibility. Antimicrob Agents Chemother 50: 1148–1155. doi: 10.1128/AAC.50.4.1148-1155.2006 16569823 10.1128/AAC.50.4.1148-1155.2006 PMC1426986

[23] Pfaller MA, Messer SA, Woosley LN, Jones RN, Castanheira M (2013) Echinocandin and triazole antifungal susceptibility profiles for clinical opportunistic yeast and mold isolates collected from 2010 to 2011: application of new CLSI clinical breakpoints and epidemiological cutoff values for characterization of geographic and temporal trends of antifungal resistance. J Clin Microbiol 51: 2571–2581. doi: 10.1128/JCM.00308-13 23720791 10.1128/JCM.00308-13 PMC3719648

[24] Bachmann SP, Patterson TF, Lopez-Ribot JL (2002) In vitro activity of caspofungin (MK-0991) against *Candida albicans* clinical isolates displaying different mechanisms of azole resistance. J Clin Microbiol 40: 2228–2230. doi: 10.1128/JCM.40.6.2228-2230.2002 12037093 10.1128/JCM.40.6.2228-2230.2002 PMC130826

[25] Cannon RD, Lamping E, Holmes AR, Niimi K, Baret PV, Keniya MV, et al (2009) Efflux-mediated antifungal drug resistance. Clin Microbiol Rev 22: 291–321, Table of Contents. doi: 10.1128/CMR.00051-08 19366916 10.1128/CMR.00051-08 PMC2668233

[26] Tsai HF, Krol AA, Sarti KE, Bennett JE (2006) *Candida glabrata PDR1*, a transcriptional regulator of a pleiotropic drug resistance network, mediates azole resistance in clinical isolates and petite mutants. Antimicrob Agents Chemother 50: 1384–1392. doi: 10.1128/AAC.50.4.1384-1392.2006 16569856 10.1128/AAC.50.4.1384-1392.2006 PMC1426987

[27] Sanglard D, Ischer F, Bille J (2001) Role of ATP-binding-cassette transporter genes in high-frequency acquisition of resistance to azole antifungals in *Candida glabrata*. Antimicrob Agents Chemother 45: 1174–1183. doi: 10.1128/AAC.45.4.1174-1183.2001 11257032 10.1128/AAC.45.4.1174-1183.2001 PMC90441

[28] Pasrija R, Panwar SL, Prasad R (2008) Multidrug transporters CaCdr1p and CaMdr1p of *Candida albicans* display different lipid specificities: both ergosterol and sphingolipids are essential for targeting of CaCdr1p to membrane rafts. Antimicrob Agents Chemother 52: 694–704. doi: 10.1128/AAC.00861-07 18056285 10.1128/AAC.00861-07 PMC2224756

[29] Mukhopadhyay K, Prasad T, Saini P, Pucadyil TJ, Chattopadhyay A, Prasad R (2004) Membrane sphingolipid-ergosterol interactions are important determinants of multidrug resistance in *Candida albicans*. Antimicrob Agents Chemother 48: 1778–1787. doi: 10.1128/AAC.48.5.1778-1787.2004 15105135 10.1128/AAC.48.5.1778-1787.2004 PMC400589

[30] Healey KR, Katiyar SK, Raj S, Edlind TD (2012) CRS-MIS in *Candida glabrata*: sphingolipids modulate echinocandin-Fks interaction. Mol Microbiol 86: 303–313. doi: 10.1111/j.1365-2958.2012.08194.x 22909030 10.1111/j.1365-2958.2012.08194.x PMC3472958

[31] Healey KR, Challa KK, Edlind TD, Katiyar SK (2015) Sphingolipids mediate differential echinocandin susceptibility in *Candida albicans* and *Aspergillus nidulans*. Antimicrob Agents Chemother 59: 3377–3384. doi: 10.1128/AAC.04667-14 25824222 10.1128/AAC.04667-14 PMC4432167

[32] Malloy PJ, Zhao X, Madani ND, Feldman D (1993) Cloning and expression of the gene from *Candida albicans* that encodes a high-affinity corticosteroid-binding protein. Proc Natl Acad Sci U S A 90: 1902–1906. 8446606 10.1073/pnas.90.5.1902 PMC45988

[33] Joets J, Pousset D, Marcireau C, Karst F (1996) Characterization of the *Saccharomyces cerevisiae FMS1* gene related to *Candida albicans* corticosteroid-binding protein 1. Curr Genet 30: 115–120. 8660467 10.1007/s002940050109

[34] Silva LV, Sanguinetti M, Vandeputte P, Torelli R, Rochat B, Sanglard D (2013) Milbemycins: more than efflux inhibitors for fungal pathogens. Antimicrob Agents Chemother 57: 873–886. doi: 10.1128/AAC.02040-12 23208712 10.1128/AAC.02040-12 PMC3553706

[35] Basso LR Jr., Gast CE, Mao Y, Wong B (2010) Fluconazole transport into *Candida albicans* secretory vesicles by the membrane proteins Cdr1p, Cdr2p, and Mdr1p. Eukaryot Cell 9: 960–970. doi: 10.1128/EC.00355-09 20348384 10.1128/EC.00355-09 PMC2901649

[36] Pinto e Silva AT, Costa-de-Oliveira S, Silva-Dias A, Pina-Vaz C, Rodrigues AG (2009) Dynamics of in vitro acquisition of resistance by *Candida parapsilosis* to different azoles. FEMS Yeast Res 9: 626–633. doi: 10.1111/j.1567-1364.2009.00508.x 19385998 10.1111/j.1567-1364.2009.00508.x

[37] Guinea J, Sanchez-Somolinos M, Cuevas O, Pelaez T, Bouza E (2006) Fluconazole resistance mechanisms in *Candida krusei*: the contribution of efflux-pumps. Med Mycol 44: 575–578. doi: 10.1080/13693780600561544 16966178 10.1080/13693780600561544

[38] Prudencio C, Sansonetty F, Sousa MJ, Corte-Real M, Leao C (2000) Rapid detection of efflux pumps and their relation with drug resistance in yeast cells. Cytometry 39: 26–35. 10655560 10.1002/(sici)1097-0320(20000101)39:1<26::aid-cyto5>3.0.co;2-c

[39] Dujon B, Sherman D, Fischer G, Durrens P, Casaregola S, Lafontaine I, et al (2004) Genome evolution in yeasts. Nature 430: 35–44. doi: 10.1038/nature02579 15229592 10.1038/nature02579

[40] Kitada K, Yamaguchi E, Arisawa M (1995) Cloning of the *Candida glabrata TRP1* and *HIS3* genes, and construction of their disruptant strains by sequential integrative transformation. Gene 165: 203–206. 8522176 10.1016/0378-1119(95)00552-h

[41] Ueno K, Uno J, Nakayama H, Sasamoto K, Mikami Y, Chibana H (2007) Development of a highly efficient gene targeting system induced by transient repression of *YKU80* expression in *Candida glabrata*. Eukaryot Cell 6: 1239–1247. doi: 10.1128/EC.00414-06 17513567 10.1128/EC.00414-06 PMC1951112

[42] Jones T, Federspiel NA, Chibana H, Dungan J, Kalman S, Magee BB, et al (2004) The diploid genome sequence of *Candida albicans*. Proc Natl Acad Sci U S A 101: 7329–7334. doi: 10.1073/pnas.0401648101 15123810 10.1073/pnas.0401648101 PMC409918

